# Implication for Functions of the Ectopic Adipocyte Copper Amine Oxidase (AOC3) from Purified Enzyme and Cell-Based Kinetic Studies

**DOI:** 10.1371/journal.pone.0029270

**Published:** 2012-01-04

**Authors:** Sam H. Shen, Diana L. Wertz, Judith P. Klinman

**Affiliations:** 1 Department of Chemistry, University of California, Berkeley, California, United States of America; 2 Department of Molecular and Cell Biology and the California Institute for Quantitative Biosciences, University of California, Berkeley, California, United States of America; University Paris Diderot-Paris 7, France

## Abstract

AOC3 is highly expressed in adipocytes and smooth muscle cells, but its function in these cells is currently unknown. The *in vivo* substrate(s) of AOC3 is/are also unknown, but could provide an invaluable clue to the enzyme's function. Expression of untagged, soluble human AOC3 in insect cells provides a relatively simple means of obtaining pure enzyme. Characterization of enzyme indicates a 6% titer for the active site 2,4,5-trihydroxyphenylalanine quinone (TPQ) cofactor and corrected *k*_cat_ values as high as 7 s^−1^. Substrate kinetic profiling shows that the enzyme accepts a variety of primary amines with different chemical features, including nonphysiological branched-chain and aliphatic amines, with measured *k*_cat_/K_m_ values between 10^2^ and 10^4^ M^−1^ s^−1^. K_m_(O_2_) approximates the partial pressure of oxygen found in the interstitial space. Comparison of the properties of purified murine to human enzyme indicates *k*_cat_/K_m_ values that are within 3 to 4-fold, with the exception of methylamine and aminoacetone that are ca. 10-fold more active with human AOC3. With drug development efforts investigating AOC3 as an anti-inflammatory target, these studies suggest that caution is called for when screening the efficacy of inhibitors designed against human enzymes in non-transgenic mouse models. Differentiated murine 3T3-L1 adipocytes show a uniform distribution of AOC3 on the cell surface and whole cell K_m_ values that are reasonably close to values measured using purified enzymes. The latter studies support a relevance of the kinetic parameters measured with isolated AOC3 variants to adipocyte function. From our studies, a number of possible substrates with relatively high *k*_cat_/K_m_ have been discovered, including dopamine and cysteamine, which may implicate a role for adipocyte AOC3 in insulin-signaling and fatty acid metabolism, respectively. Finally, the demonstrated AOC3 turnover of primary amines that are non-native to human tissue suggests possible roles for the adipocyte enzyme in subcutaneous bacterial infiltration and obesity.

## Introduction

Copper amine oxidases (CAOs) catalyze the oxidative deamination of primary amines to form hydrogen peroxide, ammonia, and the corresponding aldehyde, eq 1:
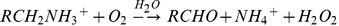
(1)These enzymes were first found to contain a novel redox cofactor, the tyrosyl-derived topaquinone (TPQ) in the bovine serum amine oxidase [1], with the distribution of TPQ now assigned to all living aerobic organisms including bacteria, yeast, plants, and animals with few exceptions such as the laboratory strain of *Saccharomyces cerevisiae*. The susceptibility of TPQ to carbonyl reagents, such as semicarbazide, has given rise to an alternate designation of membrane-associated CAOs as a semicarbazide-sensitive amine oxidase (SSAO) [2]. Efforts to elucidate the reaction mechanism of CAOs have resulted in a consensus ping-pong mechanism during which reducing equivalents from the amine substrate are stored in an aminoquinol form of cofactor, which undergoes recycling to TPQ concomitant with the conversion of O_2_ to H_2_O_2_ [3].

Though much is known about the enzymatic mechanism of CAOs, the physiological role of mammalian enzymes is not well understood, though it has been associated with several diseases. Among the three TPQ-containing CAOs annotated in the human genome (AOC1, 2 and 3), AOC3 has been implicated in congestive heart failure [4], diabetes [5], Alzheimer's [6], liver disorders [7], and cancer [8]. An understanding of the physiological role of AOC3, alternatively referred to as vascular adhesion protein-1 (VAP-1), may provide a basis for treatment of these diseases.

In bacteria and yeast, the CAOs are generally assumed to catalyze the release of nitrogen and carbon from primary amines for the support of microbial growth. Interestingly, CAOs are among the most abundant soluble proteins found in the extracellular fluids of pea, lentil, and chickpea seedlings and are implicated in wound repair [9]. In mammals, the physiological role of CAOs was initially thought to involve the metabolism of xenobiotic and endogenously produced amines. However, the natural substrate(s) of AOC3 *in vivo* is/are currently unknown and kinetic data with purified enzyme have been sparse.

The distribution of AOC3 in mammalian tissues is wide-ranging with relatively high expression shown in smooth muscle cells and adipocytes [10]. In fact, it was found to contribute up to 2.3% of total plasma membrane proteins in rat adipocytes [11]. To complicate matters further, AOC3 is not only localized to the extracellular surface of cells, known as membrane-bound AOC3, but also exists as a soluble enzyme in plasma [12] and it is unknown whether membrane-bound and plasma AOC3 have similar physiological roles. Notably, endothelial AOC3 has been implicated in the extravasation of leukocytes into inflamed tissue, acting as an adhesion protein [13]. The process of extravasation requires chemokines, cytokines, and an array of adhesion molecules [14]. It has been shown that the enzymatic activity of AOC3 is functionally important, impairing leukocyte recruitment if activity is abolished either by inhibition or site-directed mutagenesis [15]. Interestingly, a lectin, Siglec-10, expressed by leukocytes was found to be a possible substrate of AOC3 and may be involved in mediating adhesion [16], though the proposed oxidation of an arginine side chain seems highly unlikely. Currently, the precise mechanism of how AOC3 acts as a vascular adhesion protein is unknown.

With the rise in worldwide rates of obesity, type-2 diabetes, and metabolic syndrome X, interest in the biology of adipocytes has increased, especially after the discovery of a class of more than fifty adipose-derived cytokines, or adipokines [17]. Rather than functioning as a passive organ primarily involved in fat storage, insulation, and protection, adipose tissue is now thought to be involved in a complex network of endocrine, paracrine, and autocrine signals that influences the functions of many tissues [18]. AOC3 is not thought to function as an adhesion protein in adipocytes and the function of this highly expressed, extracellular enzyme is currently unknown.

Since adipose tissue plays an integral role in energy balance, a possible role of adipocyte AOC3 could be an involvement in insulin signaling. When rodents were administered AOC3 substrates such as methylamine, through dietary supplementation, they showed improvement in glucose tolerance [19]. However, co-administration of the non-physiological vanadate was required to observe a pronounced effect, which can be rationalized by a vanadate-dependent inhibition of tyrosine phosphatase or stimulation of tyrosine protein kinases [20]. Interestingly, administration of AOC3 substrate in the presence of catalase effectively abolished the insulin-sensitizing effects, implicating an important role for H_2_O_2_ [21]. Hydrogen peroxide has increasingly gained recognition as a possible cellular signaling molecule and is thought to play a role in cell proliferation, differentiation, migration, and apoptosis [22], [23]. Specifically in adipocytes, H_2_O_2_ has been shown to be involved in the activation of the insulin-signaling cascade [24]. It has also been found that long-term exposure of obese rats to the AOC3 inhibitor, semicarbazide, decreased fat deposition due most likely to enhanced lipolysis, though decreased food intake was also observed [25]. Though there may be a link between AOC3 and insulin-signaling, there have been no other reports with regards to how AOC3 may be involved in the insulin-signaling cascade and the need for vanadate to produce a pronounced effect makes this link somewhat tenuous.

With an interest in understanding the physiological function of AOC3 in adipocytes, we have focused on characterizing the suitability of various amine substrates, including primary amines annotated in the Human Metabolome database [26], for turnover by measuring kinetic parameters using the cloned human AOC3 expressed and purified from insect cells. Since animal studies most often use mouse models, a comparison of human to mouse enzyme is also reported. Finally, differentiation of murine-derived fibroblasts to adipocytes permits a comparison of purified enzyme to cell-associated AOC3. Based on the resulting profile of substrate specificity, a number of formerly unrecognized substrates and possible biological functions emerge.

## Materials and Methods

All chemicals, reagents, and column chromatographic resins, including AOC3 substrates, were purchased from Sigma Aldrich (St. Louis, MO) unless otherwise stated. Aminoacetone was purchased from Tyger Scientific, Inc. (Ewing, NJ). [1,1-^2^H_2_]Benzylamine hydrochloride was prepared as previously described [27]. The purity of synthesized [1,1-^2^H_2_]benzylamine hydrochloride was verified by NMR with no evidence of contamination by the protium substrate or other chemical contaminants. Peptide-bound lysine (GGGGKGGGG) was synthesized by the Stanford University Protein and Nucleic Acid Facility (PAN). Calcium phosphate transfection kit was purchased from Invitrogen (Carlsbad, CA). DMEM High Glucose, purchased from GIBCO (Carlsbad, CA) was used in the course of culturing adipocytes. Gel filtration column chromatographic resin, Sephacryl S-200 HR, was purchased from Amersham Biosciences (Piscataway, NJ).

### Cell Culture

s2 *Drosophila* cells were purchased from Invitrogen (Carlsbad, CA) and grown in BioWhittaker Insect-XPRESS insect cell media from Lonza (Walkersville, MD) at 27°C. For antibiotic selection, s2 media was supplemented with 600 ug/mL hygromycin purchased from Invitrogen (Carlsbad, CA). Murine 3T3-L1 preadipocytes were obtained from the American Type Cell Culture (Bethesda, MD). The adipocyte differentiation protocol starts with a 100 mm tissue culture dish of 3T3-L1 preadipocytes grown to confluence. Once confluent, cellular differentiation was induced using insulin, dexamethasone, and methylisobutylxanthine as previously described [28]. Cells were cultured in Dulbecco's Modified Eagle Medium (DMEM) supplemented with 10% fetal bovine serum (FBS) purchased from Hyclone (Waltham, MA). Only passage 1 or 2 adipocytes were used at Days 8 to 10 post-differentiation. Adipocytes were maintained at 37°C and 5% CO_2_.

### Construction of Expression Vector for Producing Soluble Human and Murine AOC3

The soluble portion of human AOC3 (GenBank Accession No. NM_003734) from residues 28 to 763 was cloned by PCR from a VAP-1 (vascular adhesion protein-1, AOC3) cDNA-clone plasmid (generously provided by Prof. Sirpa Jalkanen) containing the complete coding sequence of human AOC3. Amplification was performed using the 5′ primer 5′-TATCAG**GAATTC** ATGCAGGGGTGGAGATGGGGGTGA-3′ with EcoRI restriction site introduced upstream of the N-terminus. The 3′ primer was 5′-TATCAG **CTCGAG**TTACTAGTTGTGAGAGAAGCCCCCGTG-3′ with XhoI restriction site introduced after two stop codons. PCR was carried out for 30 cycles with 1 min denaturing at 95°C, 1 min annealing at 60°C, and 2 min 15 sec elongation at 72°C. The PCR product was ligated into the s2 expression vector pMT/BiP/V5-His B from Invitrogen (Carlsbad, CA). The extra 17 amino acids at the N-terminus of recombinant human AOC3 was due to the restriction enzymes used. Upstream restriction enzymes were not used due to the presence of cut sites within AOC3. For murine AOC3 (clone also generously provided by Prof. Sirpa Jalkanen), a construct was created in a manner similar to the human AOC3 in which the first 27 amino acids corresponding to the membrane anchor were removed. PCR was carried out on the full-length AOC3 sequence with the following primers: 5′ primer 5′-GTG**GAATTC**ATGGGCAGGAGCGGAGATG-3′ and 3′ primer, 5′-ATA**TCTAGA**TACAGTT CAATTGTCTCTGTAAGCAAAG-3′. The trun-cated sequence was inserted into the pMT/BIP/V5-His B vector using the EcoRI and XbaI restriction sites.

### Transfection of Schneider 2 (s2) Drosophila Cells and Selection of Stable Cell Line

s2 cells were grown to 2 to 4×10^6^ cells/mL in serum-free insect cell media and co-transfected with pMT/BiP/V5-His B-human AOC3 plasmid and pCoHygro selection vector using calcium phosphate. The suggested transfection procedure provided by the manufacturer was followed except calcium chloride and phosphate solutions were added 10 uL at a time with ample mixing. Two days post-transfection, s2 cells were washed twice and resuspended in cell media supplemented with 10% FBS and 300 ug/mL hygromycin. The s2/hygromycin kill curve was determined indicating that a minimum dose of 600 ug/mL hygromycin was required to kill all untransfected cells. After one week, antibiotic dose was increased to 600 ug/mL hygromycin and selection continued for two more weeks. Amine oxidase activity was not detectable in small-scale test expressions of 50 to 100 mL. Samples showing a band around 90 kDa on SDS-PAGE gel were scaled up to 500 mL total volume and eluted from an ion exchange column prior to assaying for activity (see below). Transfection with the murine AOC3 plasmid used protocols similar to those for human AOC3.

### Western Blot Analysis

Samples were run on 10% acrylamide, denaturing SDS-PAGE gel using a Laemmli buffering system and transferred to nitrocellulose membrane. Blocking was performed for 1 h at room temperature. After washing three times, membrane was incubated with a 1∶2400 dilution of primary anti-human or murine AOC3 antibody (generously provided by Prof. Sirpa Jalkanen) in blocking buffer with gentle agitation for approximately 14 to 16 h at 4°C. Membrane was then washed three times and incubated in 1∶1000 dilution of secondary antibody, horseradish peroxidase conjugated anti-mouse Ig from Cell Signaling Technology (Danvers, MA) with gentle agitation for 1 h at room temperature. The membrane was immediately developed using ECL Plus Western Blotting Detection System from GE Healthcare, (Buckinghamshire, UK).

### Purification of Human AOC3

All steps of purification were performed at 4°C and were initiated immediately post-expression. Starting with 1.5 L raw expression media, the s2 cells were spun down at 4000 rpm for 15 min. The supernatant was isolated and concentrated to approximately 750 mL. The concentrated media was then dialyzed in 12 L of 10 mM potassium phosphate (KPi) buffer, pH 6.5 for at least 4 h, after which buffer was replaced and dialysis allowed to continue for approximately 15 h more. The first purification step involved cation exchange chromatography using 75 mL of SP Sepharose Fast Flow equilibrated with 10 mM KPi, pH 6.5. The dialyzed media was filtered (0.22 um) and loaded onto the cation exchange column and eluted off the column with 1 L buffer step gradient (500 mL 10 mM KPi pH 6.5 and 500 mL 10 mM KPi pH 6.5/250 mM NaCl) into fractions by gravity. The fractions were analyzed on a SDS-PAGE gel and those containing AOC3 were collected and pooled. The pooled fractions were concentrated to approximately 10 mL and diluted with 10 mL 10 mM KPi buffer, pH 6.5, 20% glycerol to make a final 20 mL 10% glycerol solution to help stabilize the protein and minimize precipitation. The glycerol did not affect enzyme activity when compared to activity of enzyme purified without addition of glycerol. The 20 mL partially purified protein was further concentrated down to 1 to 2 mL. The concentrate was centrifuged and the soluble portion loaded onto a gel filtration column, charged with 290 mL Sephacryl S-200 HR equilibrated with 50 mM KPi, pH 6.5. A pump was connected to the gel filtration column with flow regulator, collecting 1 mL fractions over 16 to 20 min. Fractions were run on a SDS-PAGE gel and those indicating purified AOC3 were collected and concentrated to approximately 50 to 200 uL. Benzylamine oxidase activity was measured. The benzylamine substrate was dissolved in 50 mM KPi, pH 6.5 at a concentration of 2 mM. Buffer with benzylamine was blanked in a Cary 50 Bio UV-Vis spectrophotometer from Varian (Palo Alto, CA) before addition of either 3 uL or 6 uL purified AOC3 to initiate reaction at room temperature and total volume of 110 uL. Benzaldehyde product formation was monitored at absorbance λ = 250 nm (ε = 13800 M^−1^ cm^−1^ [29]) for 1 min. Purified protein was immediately snap-frozen in 50 mM KPi, pH 6.5 buffer with liquid nitrogen and stored at −20°C. The purification of murine AOC3 was performed in a similar manner to human AOC3, with minor modifications. Murine AOC3 was purified using anion exchange resin, DEAE Sepharose Fast Flow from Amersham Biosciences (Piscataway, NJ), equilibrated with 5 mM KPi, pH 7.2, eluting with two 1 L buffer gradients composed of either equal volumes of 5 mM KPi, pH 7.2 and 100 mM KPi, pH 7.2 or 100 mM KPi, pH 7.2 and 300 mM KPi, pH 7.2. Enzyme mostly eluted off the anion exchange column after the 5 mM/100 mM gradient. Gel filtration conditions were similar, though collected protein was loaded onto the same column 2 to 3 times.

### Determination of TPQ Content

The TPQ content of AOC3 was determined using phenylhydrazine in 50 mM KPi, pH 6.5 [30], at room temperature by measurement of the change in absorbance at λ = 448 nm with and without phenylhydrazine using ε = 40500 M^−1^ cm^−1^. This extinction coefficient was determined for copper amine oxidase expressed by the yeast, *Hansenula polymorpha* (cf. [3]). The concentration of enzyme monomer was determined by Bradford assay using reagents from Biorad (Hercules, CA) using bovine albumin, fraction V from Pierce (Rockford, IL) standard and MW per monomer of 84622.

### ICP

Copper and zinc standards were made through serial dilutions from reference solutions from Fisher Scientific (Pittsburgh, PA). ICP was performed on a Perkin Elmer Optima 7000 (Waltham, MA).

### Kinetic Characterization of AOC3

Steady-state kinetic measurements were carried out by monitoring oxygen consumption using a Clark electrode and YSI Model 5300 Biological Oxygen Monitor. In the case of human AOC3, a final volume of 1 ml contained 500 mM KPi, pH 7.4 at 37°C, to which variable amounts of substrate were added; reaction was initiated by addition of AOC3. For the screening of murine AOC3, the conditions were similar to human AOC3, although the concentration of buffer was only 50 mM. Unless otherwise noted, the oxygen concentration was kept constant at 211 µM. Data were fitted to the Michaelis-Menten equation, and *k*_cat_ was calculated using the active protein concentration as determined by phenylhydrazine assay described above.

### Microscopic Examination of the Subcellular Localization of Murine AOC3

3T3-L1 preadipocytes were grown and differentiated on glass coverslips coated with poly-L-lysine (MW = 100K–150K). On day 5 post-differentiation, cell media was replaced with fresh DMEM containing 10% FBS. Cells were refrigerated prior to use. Mature adipocytes on coverslips were prepared for immunofluorescence by washing twice with PBS. Cells were fixed by incubating with 4% paraformaldehyde for 10 min [31]. After blocking, cells were incubated with a monoclonal primary anti-mouse AOC3 antibody followed by the secondary anti-rat Ig antibody [32]. After washing with phosphate-buffered saline (PBS), cells were mounted using Prolong Kit from Molecular Probes (Carlsbad, CA). Cells were allowed to sit overnight before evaluation by confocal microscopy.

### Whole Cell Assay of Murine AOC3 Activities

Murine 3T3-L1 adipocytes were cultured in 24-well plates. Prior to the experiment, cells were washed twice with warmed PBS and once with warmed DMEM+10% FBS. Only wells fully populated by differentiated adipocytes (at least 90% of cell population) were used. For measurements involving isoamylamine substrate, adipocytes were incubated in 500 uL DMEM+10% FBS and all cells treated with 0.33 mM clorgyline and 3.33 mM L-deprenyl to eliminate activity of monoamine oxidase from measurements [33]. In the set of control experiments, adipocytes were also treated with 1 mM AOC3 inhibitor, semicarbazide. Cells were incubated with inhibitors for 30 min at 37°C and 5% CO_2_. After incubation, cells were washed twice with warmed Krebs Ringer Phosphate (KRP) (145 mM NaCl, 5.7 mM sodium phosphate, 4.86 mM KCl, 0.54 mM CaCl_2_, 1.22 mM MgSO_4_, 5.5 mM glucose, pH 7.35) buffer. A coupled enzymatic reaction involving horseradish peroxidase (HRP) and Amplex Red purchased from Molecular Probes (Carlsbad, CA) was used to detect H_2_O_2_ production resulting from substrate turnover. Upon final addition of 500 uL KRP buffer containing 50 uM Amplex Red reagent and 0.1 U/mL HRP, isoamylamine (from 125 uM to 8 mM) was added to wells pre-incubated either with or without inhibitor. All procedures involving Amplex Red were performed in the dark. Envision Multilabel Reader with a 570 nm optical filter from Perkin Elmer (Waltham, MA) was used to determine the absorbance of oxidized Amplex Red. Measurements were taken every 3 min for 30 min at 37°C. Endpoint absorbance changes were used to determine kinetic rate and to calculate the whole cell K_m_ of isoamylamine.

For measurements involving aminoacetone and methylamine, mature adipocytes were washed twice with room temperature phosphate buffered saline followed by incubation in 1 ml Krebs Ringer phosphate glucose solution for 30 min. One unit (1 U) of horseradish peroxidase and 50 ng of Amplex Red dye were added to each well. After mixing, the plate was incubated at 37°C in a fluorescence plate reader (Gemini System using the Softmax software package) and a background rate for Amplex Red oxidation was measured (λ_Ex_ = 560 nm and λ_Em_ = 590 nm). Aminoacetone or methylamine was then added and the rate of Amplex Red oxidation was determined by subtracting the background from the rate obtained after substrate addition. Both direct (slope) and endpoint fluorescence changes were measured and the determined rates were used to calculate an averaged K_m_ value.

## Results

### Untagged, Recombinant Human AOC3

Previous recombinant forms of human AOC3 have been purified via conjugation of a modified GST tag to the N-terminus of the soluble enzyme and the concomitant use of glutathione-affinity chromatography [34], immunoaffinity chromatography using a N-terminal FLAG epitope [35], or a one-step purification using commercially unavailable anti-AOC3 monoclonal antibody [36]. Herein, a purification procedure of the untagged soluble human AOC3 enzyme was developed. Attempts to express recombinant AOC3 in *E. coli* and *S. cerevisiae* were unsuccessful. Expression proved viable in s2 *Drosophila* cells, consistent with the earlier report by Dooley and co-workers of the overexpression of human kidney diamine oxidase (AOC1) in the s2 insect cell line [37].

**Figure 1:**
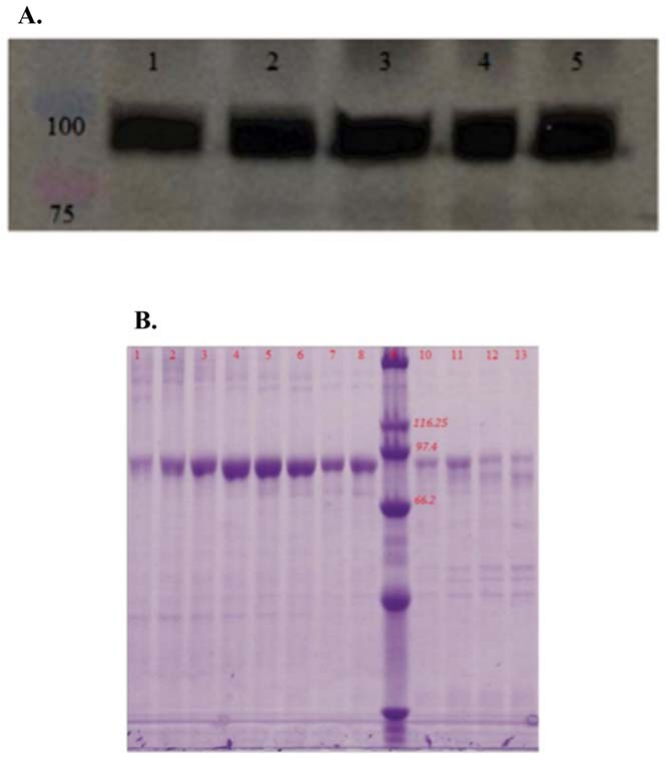
Purification of human AOC3 expressed by s2 *Drosophila* cells. A. Immunoblot of fractions obtained after ion exchange column chromatography, showing human AOC3 at the expected mass. Protein ladder to the left of Lane 1 (top band, blue −100 kDa; bottom band, red −75 kDa). B. 10% acrylamide, denaturing SDS-PAGE gel electrophoresis of fractions using a Laemmli buffering system after gel filtration column chromatography. Fractions represented in lanes 4 to 8 and 10 were isolated and concentrated for further characterization. Protein ladder shown in Lane 9 (second band from top −116 kDa, third band −97 kDa, fourth band −66 kDa). Approximately 0.25–2 ug of protein was loaded into each lane.

In this study, the soluble portion of human AOC3 was cloned into an expression plasmid containing an upstream-inducible metallothionine promoter and BiP secretion signal and stably transfected into s2 *Drosophila* cells, resulting in secretion of the enzyme into cell media upon induction. An immunoblot of fractions from the ion exchange chromatograph resulted in a significant band at approximately 90 to 100 kDa, the expected size of the monomer of soluble AOC3 calculated as 84 kDa in the absence of posttranslational modification, Fig. 1A. Subsequent purification of the eluant from cation exchange by gel filtration chromatography resulted in pure enzyme, as shown in Fig. 1B.

The yield of enzyme varied from 160 to 480 ug/L of purified enzyme. The transfection of s2 cell lines using calcium phosphate after selection has been reported to result in up to 500 copies of inserted genes with expression of up to 50 mg/L of recombinant human enzymes reported [38], [39]. The relatively low yield of AOC3 is attributed to poor transfection efficiency. Nonetheless, the yield of AOC3 obtained in this manner is comparable to that from other expression procedures, with the advantages of untagged protein and easily accessible purification steps. The uncorrected specific activity (see below) was approximately 46 nmol/min/mg of monomer.

### Characterization of Purified Human AOC3

Observed rates were linear in enzyme concentration and eliminated by prior incubation of purified enzyme with 100 uM semicarbazide inhibitor for 5 min. The quinone moiety of the AOC3 active site cofactor, TPQ, can be derivatized using the carbonyl reagent phenylhydrazine to yield the resulting hydrazone product, which can be monitored at 448 nm. The percent of active protein determined in this manner was approximately 6.1%, Table 1, leading to a corrected specific activity of ca. 0.76 µmol/min/mg of monomer under the conditions of the standard assay. Since the biogenesis of TPQ requires copper [40], spiking purified enzyme with sub-stoichiometric amounts of CuCl_2_ could increase the percent of active protein. However, the specific activity actually decreased with precipitation observed (data not shown).

**Table 1:** Properties of purified human AOC3.

Property	Value
TPQ content^a^	0.06
Copper content^a^	0.4
K_m_ (O_2_)	28±4.6 torr 38±4.2 uM
*k_cat_*_(H)_/*k_cat_*_(D)_ b	1.23±0.08
[*k*_cat_/K_m_(S)]_H_/[*k*_cat_/K_m_(S)]_D_ b	8.64±2.82

a Per monomer.

b At ambient O_2_, pH 7.4, 37°C.

The copper content was determined by ICP and found to be 0.4 moles per mole of AOC3 monomer. The copper content is surprisingly low, given that s2 cells were induced to express AOC3 with 600 uM CuSO_4_. Additionally, the zinc content was found to be 0.16 moles per mole of AOC3 monomer. Zinc-substituted bovine serum amine oxidase has been found to be catalytically inert [41], with tighter binding of the apo-enzyme for zinc than copper shown in the yeast *H. polymorpha* amine oxidase [42]. The inactivity of Zn^2+^-reconstituted enzyme is due to the requirement for a redox active metal during TPQ biogenesis [43]. In light of only 40% of the total protein subunits containing copper, the efficiency of post-translational cofactor biogenesis is estimated as 15%.

### Steady-State Substrate Kinetic Profiling of Human AOC3

Most kinetic measurements of AOC3 have relied on the use of cell lysates or membranes, rather than pure enzyme, and colorimetric reagents, such as Amplex Red, to measure hydrogen peroxide production [44]. This approach requires the determination of any sub-cellular background reaction rate following the introduction of an AOC3 inhibitor. In addition, interaction of AOC3 with cellular components could affect enzyme activity.

Rather than measure hydrogen peroxide production, O_2_ consumption was monitored directly using a Clark oxygen electrode. A high ionic strength (500 mM potassium phosphate buffer) was maintained so that the assay of all substrates (including those with elevated K_m_ values) could be carried out at the same final ionic strength. Comparison of steady-state kinetic rates of benzylamine oxidation with results from kinetic assays at lower ionic strength (50 mM potassium phosphate buffer) did not show a significant difference, indicating little impact on rate due to the higher ionic strength for this substrate. Addition of 10 to 150 mM NaCl to kinetic assays of human copper amine oxidase activity has previously been found to have little effect on the rate-determining step [44]. Using the purified AOC3 and our O_2_ uptake assay, a variety of aryl-, straight chain alkyl-, and branched chain alkylamines, including several amines in the Human Metabolome database [26], were examined for activity with measured values of *k*_cat_ and K_m_ in Table 2.

**Table 2:** Human AOC3 substrate kinetic profile.

Substrate	*k*_cat_(s^−1^)	K_m_(uM)	*k*_cat_/K_m_(M^−1^ s^−1^)
Methylamine^a^	5.60±0.20	652±77	8.58±0.12×10^3^
Ethylamine^a^	6.12±0.40	12800±1960	4.78±0.17×10^2^
Propylamine	5.80±0.23	2650±260	2.19±0.10×10^3^
Butylamine	6.31±0.31	2830±460	2.23±0.17×10^3^
Amylamine	4.33±0.19	5710±760	7.57±0.14×10^2^
Isobutylamine	7.40±0.51	3420±540	2.16±0.17×10^3^
Isoamylamine	6.89±0.83	4560±1160	1.51±0.28×10^3^
Benzylamine	3.42±0.10	84.5±9.8	4.05±0.12×10^4^
Phenethylamine^a^	1.12±0.08	2050±620	5.45±0.31×10^2^
Cyclohexanemethylamine	5.36±0.77	16500±5200	3.25±0.35×10^2^
Dopamine^a^	0.54±0.033	99±24	5.44±0.25×10^3^
Aminoacetone^a^	1.46±0.10	66±18	2.23±0.28×10^4^
Cysteamine^a^^*,*^ b	1.11±0.14	31±15	3.55±0.50×10^4^

^*a*^Substrates of human AOC3 with entries found in the Human Metabolome Database version 2.5.

^*b*^Substrate kinetics were measured at approximately 19 to 23% air (4 to 5% O_2_).

Both methylamine and ethylamine are found endogenously in mammals [45], [46]. However, when comparing *k*_cat_ and K_m_ of straight-chain alkylamines, no apparent trend in k_cat_ and K_m_ is observed as the alkyl chain is lengthened (1 to 5 carbons). In an earlier study, a decrease in V_max_ and increase in V_max_/K_m_ were reported with an increase in chain size (up to 9 carbons) using AOC3 derived from human-solubilized adipocyte membranes [44]. K_m_ values are all observed to be in the mM range with the exception of methylamine (652 uM), that still contrasts markedly with human plasma levels of methylamine found to be on average 31.8 ng/mL (1.03 uM) [47]. Branched chain amines are not thought to occur endogenously, but both isobutylamine and isoamylamine are reasonably good substrates with mM K_m_ values. On the other hand, isopropylamine was not found to have any AOC3 activity. Substrates of human AOC3 also include arylamines and catecholamines, with the non-physiologic benzylamine exhibiting a low K_m_ value (84.5 uM) and the highest *k*_cat_/K_m_. Adding an extra methyl group to benzylamine results in an approximately 25-fold increase in K_m_ and a decrease in *k*_cat_/K_m_ of approximately two orders of magnitude, indicating that the endogenous phenethylamine [48] is an unlikely substrate.

Catecholamines were observed to consume oxygen under ambient air prior to addition of enzyme. However, lower concentrations of dopamine, epinephrine, and norepinephrine (below 1.2 mM) allowed measurement of steady-state kinetics, without a high background rate of oxygen consumption masking the rate of amine oxidation by AOC3. Dopamine was found to have a K_m_ of approximately 99 uM, which is about 12-fold less than the maximum substrate concentration used, allowing a reasonable estimate of *k*_cat_. Dopamine has a demonstrated role in adipocyte metabolism, binding to the β3-adrenoreceptor, resulting in lower glucose uptake [49]; in this context, AOC3 oxidation of dopamine may moderate ligand/receptor interactions. Neither norepinephrine nor epinephrine (a secondary amine), exhibited any reproducible AOC3 activity above background and other endogenous amines, such as tyramine, tryptamine, histamine, and octopamine were found to have either little or no AOC3 activity.

Aminoacetone, a threonine and glycine metabolite [50], and cysteamine, a breakdown product of pantethine (a coenzyme A precursor) [51], are both found in humans and were found to have among the lowest K_m_ values (66 uM and 31 uM, respectively), as well as relatively high second order rate constants. Both substrates can auto-oxidize in ambient air with cysteamine assays yielding a high background rate of oxygen consumption at all concentrations of amine tested. Thus, to characterize the steady-state kinetics of cysteamine oxidation, it was necessary to reduce the oxygen concentration to 19 to 23% air. This is around the K_m_(O_2_) of human AOC3, which was found to be approximately 28±4.6 torr (18% air) (Table 1), close to the partial pressure of oxygen in the interstitial space of tissue (ca. 20 to 40 torr [52]). While reduction in the O_2_ concentration is expected, thus, to reduce *k*_cat_ ca. 2-fold, it will not influence the comparative *k*_cat_/K_m_ value. The second order rate constants, *k*_cat_/K_m_, were within two orders of magnitude for all of the amines examined, 10^2^ to 10^4^ M^−1^ s^−1^.

The kinetic isotope effect (KIE) of amine oxidation was evaluated using benzylamine with both hydrogens at the α carbon replaced with deuterium (Table 1). The isotope effects were found to be 1.23±0.08 at substrate saturation and 8.64±2.82 at low substrate (below K_m_). This indicates that steps leading up to and including the base-catalyzed proton abstraction from substrate are not rate-limiting at substrate saturation, whereas the C–H cleavage step becomes rate-determining at low substrate concentrations. This result can be of assistance in evaluating the impact of variations in substrate structure on catalytic efficiency (see next section below and ref [53]). After the completion of this work, Heuts et al. published similar results for the isotope effect using benzylamine, with additional studies using phenethylamine and its para-substituted compounds [54]. Interestingly, they show a high KIE on *k*_cat_ for phenethylamine, indicating that for a slightly larger substrate, proton abstraction becomes rate-limiting under conditions of saturation by substrate and O_2_.

The mechanism of vascular adhesion by leukocytes to AOC3-expressing endothelial cells has been hypothesized to proceed via the interaction of a peptide-bound lysine on the extracellular surface of leukocytes with AOC3 [55]. This could involve either simple binding/inhibition or oxidation of the lysyl ε-amino group. In this study, neither L-lysine (minimal activity at 38 mM) nor D-lysine was found to have appreciable AOC3 activity. Similar results were shown by previous kinetic studies using bovine AOC3, though L-lysine has been shown to act as an AOC3 inhibitor, but only in the presence of benzylamine [56]. In addition, the small lysine containing peptide, GGGGKGGGG, which has been shown to be an AOC3 inhibitor [57], was found to be inactive toward AOC3 as a substrate at concentrations up to 38 mM. The ability of a protein-bound lysine to serve as the *in vivo* substrate for AOC3 seems unlikely, though a sequence-dependent activity cannot be ruled out.

Comparing previous data obtained from cell lysates containing AOC3 to the present results, [58], the K_m_ of methylamine was found to be 670 uM versus our finding of 652 uM and the K_m_ of phenethylamine was found to be 1940 uM versus our finding of 2050 uM. The K_m_ of benzylamine, determined with crude membranes from human adipose tissue [59], was found to be 175 uM versus our finding of 84.5 uM. Since the earlier data did not rely on purified enzyme, V_max_ values were reported in rate per mg of cell lysate, precluding quantification of *k*_cat_ values.

### Comparison of Human and Mouse AOC3 Substrate Kinetics

The implication of AOC3 in inflammatory disorders has made the search for small molecule inhibitors an active area of research [60]. In order to assess drug efficacy and safety, it is expected that initial trials will occur in mouse subjects. Though human and mouse AOC3 are 83% identical and 91% similar (Fig. S1), differences in the enzymatic activity may arise. To investigate these kinetic differences, the mouse AOC3 was expressed and purified as described for human AOC3 with modifications (see Experimental Procedures), and steady-state kinetics performed using a Clark O_2_ electrode. We chose to focus comparisons on the parameter *k*_cat_/K_m_ (Table 3), since the deuterium isotope effects for the human AOC3 (Table 1) indicate that this is the parameter that will be sensitive to substrate structure. We note that, analogous to human AOC3, no activity was observed with either free lysine or the lysine containing GGGGKGGGG. In the case of the methyl ester of lysine, *k*_cat_/K_m_ was ca. 12 M^−1^ s^−1^. It can be seen that for the majority of substrates examined, *k*_cat_/K_m_ varies within approximately one order of magnitude and rates are within a factor of 3 to 4 times of one another. For alkylamines, as the chain length increased, the enzymatic efficiency of human AOC3 decreases relative to the mouse enzyme, though the human enzyme is better at catalytic turnover until amylamine. For branched chained amines, the mouse enzyme was found to be more efficient; in the case of isopropylamine, neither the human nor mouse enzyme exhibited appreciable activity. Two notable differences are the substrates, methylamine and aminoacetone, that appear 10 to 12 times more active with the human AOC3. These results suggest that caution is called for when screening the efficacy of inhibitors designed against human enzymes in non-transgenic mouse models during pre-clinical analysis. In addition, the expected differences in tissue levels of AOC3 substrates in human versus mouse (see below) could greatly impact the experimental output. Future modeling studies will address structural origins of kinetic parameter differences between the murine and human enzymes, Table 3.

**Table 3:** Comparison of second order rate constants (*k*_cat_/K_m_) between purified human and mouse AOC3.

Substrate	Human^a^	Mouse^a^	Human/Mouse
Methylamine	8.58	0.730	11.8
Ethylamine	0.478	0.109	4.4
Propylamine	2.19	0.722	3.0
Butylamine	2.23	1.41	1.6
Amylamine	0.757	1.39	0.55
Isobutyamine	2.16	2.27	0.95
Isoamylamine	1.51	4.91	0.31
Benzylamine	40.5	12.9	3.2
Phenethylamine	0.545	0.955	0.57
Aminoacetone	22.3	2.94	7.6
Dopamine	5.44	18.3	0.3

^*a*^ *k*_cat_/K_m_ (M^−1^ s^−1^)×10^3^ for human and mouse AOC3.

### Murine 3T3-L1 Adipocytes Express Active Extracellular AOC3

**Figure 2:**
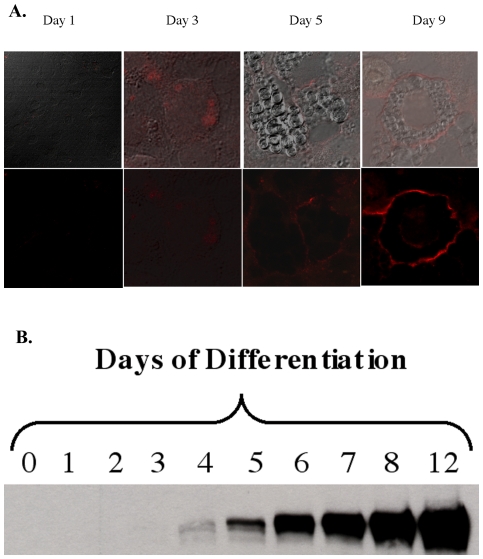
Visualization of AOC3 during murine 3T3-L1 adipocyte differentiation. A. Use of confocal fluorescence microscopy. Top, phase contrast image of adipocytes; bottom, overlay of immunofluorescence with phase contrast image (Anti-mouse AOC3 antibody courtesy of Sirpa Jalkanen). B. Differentiation, as detected by Western blotting using anti-mouse AOC3 antibody.

AOC3 expression has been shown on the extracellular surface of adipose tissue in laboratory mice [61]. In addition, AOC3, present in intracellular vesicles of endothelial cells under normal conditions, is transported to the surface during inflammation, at which point AOC3 becomes a membrane-bound ecto-enzyme [12]. Analogous experiments are necessary in cultured 3T3-L1 adipocytes, to validate *in vitro* assays. AOC3 has been found to be absent in undifferentiated Day 0 3T3-L1 pre-adipocytes and abundant in Day 8 mature lipid-laden adipocytes [62]. In addition, AOC3 activity is thought to ameliorate insulin resistance and is implicated in insulin signaling [20], [21]. If AOC3 plays an important role in insulin signaling, it is possible that the enzyme may co-localize with the insulin receptor, which is purportedly found in lipid raft domains prevalent in adipocytes and known as caveolae [63]. However, the localization of AOC3 expression is found to be uniform, covering the extracellular surface of Day 9 adipocytes as shown by immunofluorescence in Fig. 2A, making co-localization unlikely, at least under *ex vivo* cell culture conditions. AOC3 expression was not prevalent until Day 5 during the differentiation process and continued to increase at Day 12 as shown by the immunoblot in Fig. 2B. Plasma membrane AOC3 is abundant on the extracellular surface of mature 3T3-L1 adipocytes, though entirely absent in pre-adipocytes.

**Figure 3:**
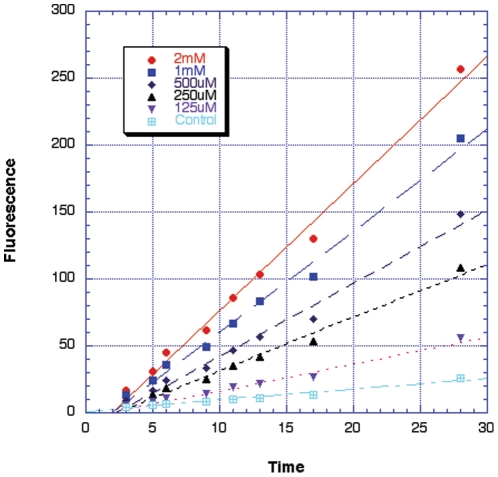
Adipocyte cell culture-based determination of AOC3 turnover in the presence of varying concentrations of methylamine substrate using the Amplex Red peroxide detection assay.

With experimental verification of the extracellular expression of AOC3 by 3T3-L1 adipocytes, we moved to an examination of the enzyme in its cellular context. Agreement between purified enzyme and whole cells would support the utilization of data from isolated enzyme in making conjectures regarding possible *in vivo* substrates. Kinetic analyses were performed using Amplex Red dye, since direct oxygen uptake assays were expected to be complicated by other cellular processes. Figure 3 shows the time concentration dependence of extracellular H_2_O_2_ production with methylamine as substrate, demonstrating a linear increase in peroxide production that is dependent on varying substrate concentrations. In Table 4, K_m_ values are summarized with no effort in the present study to obtain the total AOC3 concentration on the surface of adipocytes as a prerequisite for *k*_cat_. The K_m_ values are within 1.5 to 4.2-fold of values determined with purified murine AOC3. Further, the K_m_ values for methylamine and aminoacetone with human AOC3 lie between the K_m_ values determined with either the purified murine enzyme or murine-derived adipocytes. The obvious outlier in Table 4 is the 10-fold larger K_m_ for isoamylamine toward purified human enzyme in relation to murine adipocyte-associated AOC3. The rate of hydrogen peroxide production by the murine 3T3-L1 adipocytes in the presence of 750 uM isoamylamine was quantified to be ca. 11 uM/h per million cells, a level that has been shown to induce oxidative stress signaling in mammalian cells [64].

**Table 4:** Comparison of AOC3 K_m_ values determined by steady state kinetic studies of purified murine enzyme and whole cell 3T3-L1 adipocytes.

Substrate	Murine3T3 cells (uM)	Purified murine AOC3 (uM)	Purified human AOC3 (uM)
Methylamine	513^b^	800	652
Aminoacetone	35^b^	150	66
Isoamylamine	432^a^	150	4560

^*a*^K_m_ value calculated by plotting endpoint rates vs. substrate concentration, correcting for baseline Amplex Red oxidation by adipocytes pre-treated with 1 mM semicarbazide for 30 min.

^*b*^K_m_ values are averages derived from both endpoint fluorescence versus substrate concentration and linear rates of Amplex Red oxidation versus substrate concentration.

## Discussion

A very puzzling finding - that the identical CAO (designated AOC3) appears on the outer plasma membrane of both human endothelial tissue and the human adipocyte [65] - was one of the initial motivations behind the present study. Whereas an elegant series of studies by Jalkanen and co-workers has demonstrated the role of the endothelial AOC3 in the recruitment and internalization of leukocytes during an inflammatory response [13], a similar primary role for the adipocyte-associated AOC3 has appeared unlikely. In fact, prior to the present study, the most likely link of the adipocyte AOC3 to cellular metabolism was an enhancement of insulin-stimulated glucose uptake [19], [21] via an unidentified pathway.

The approach taken herein has involved a combination of studies using either human or murine-derived materials. A new expression system for human AOC3 has been developed that involves the removal of the 27 amino acid N-terminal transmembrane domain and expression in s2 *Drosophila* cells. Pure protein is obtained without using peptide-tagged enzyme or reliance on proprietary monoclonal antibody (Fig. 1), with a yield that is optimally ca. 0.5 mg/L. Better enzyme yields likely was deterred by unmatched codon bias between a human-encoded mRNA and insect-optimized translational machinery [66]. However, it is possible that future optimization of expression conditions may increase this yield significantly, given previously demonstrated examples of quite high level expression of human gene products in s2 cells [37]. We note that insect cell expression will likely result in a different pattern of AOC3 post-translational surface glycosylations, which has been shown to affect catalytic activity [67] and could impact some of our kinetic measurements of human AOC3. A second goal for the future will be to enhance the fraction of expressed AOC3 that contains its post-translationally generated cofactor, TPQ, which is currently at only 6% of total protein. Application of a similar expression system for the murine AOC3 has allowed a comparison of kinetic parameters at pH 7.4, 37°C (Table 3). While the kinetic results indicate a similarity between the murine and human AOC3 (Table 3), the use of mouse models in clinical studies aimed at moderating AOC3 activity may be fraught, especially given expected species differences in tissue levels of AOC3 substrates (see below).

Currently, the endogenous substrate(s) of AOC3 is/are unknown, including AOC3 in the endothelial context. Knowing the endogenous substrate(s) would provide an invaluable clue to the function of AOC3. However, this endeavor has many difficulties ranging from the measurement of potentially localized and low-level concentrations of primary amines *in vivo* to the likelihood of different substrates for AOC3 depending on tissue and even cell type. With this in mind, we decided to take a more general approach by generating a substrate kinetic profile based on the purified human enzyme that includes amine substrates found in the Human Metabolome database, a repository of *in vivo* metabolites. These studies, thus, provide the identity of possible endogenous substrates for future investigation. In addition, almost all previous kinetic measurements of AOC3 either utilized whole cell or crude membrane lysates in varying contexts, making comparison of results difficult. Finally, our approach employs an oxygen electrode to measure enzyme rate, and is more sensitive than the commonly used hydrogen peroxide probe, Amplex Red, which is sensitive to both photo- and hyper-oxidation. A number of molecular results have emerged regarding the properties of isolated human AOC3 enzyme that include a low K_m_ value for O_2_, in the range expected for O_2_ levels in the interstitial space of human tissue, as well as rate limitation by the chemical step of C–H abstraction under conditions of steady-state turnover of amines at concentrations below their K_m_ values. The fact that chemistry is rate-determining for *k*_cat_/K_m_ of substrate is a fairly generic property for all characterized CAOs, and may be reflective of the relatively low turnover numbers of these enzymes and their ability to act on a wide series of substrates [3]. The broad range of substrate specificity is apparent for AOC3, with both aliphatic and aromatic substrates showing turnover rates of 10^2^ to 10^4^ M^−1^ sec^−1^, following normalization of the enzyme concentration to the number of moles of TPQ per subunit (Table 2). This lack of specificity is completely consistent with the previously published active site structure of the human AOC3 [34], [35], [36] (Fig. S2) which shows a greatly expanded active site in relation to, for example, a CAO isozyme from *Hansenula polymorpha* that acts with high preference on small substrates such as methylamine [68]. Although some kinetic differences between the human and murine AOC3s are detected (Table 3), a pattern of activity on both aliphatic and aromatic amines is maintained. We note the 10-fold larger rate for the human than murine forms of enzyme is for oxidation of methylamine and aminoacetone, two primary amines shown to be present in human tissue [69]. While several endogenous pathways for the production of methylamine are known in humans [45], [70], the pathway for aminoacetone production is less clear. Aminoacetone is generally formed predominantly via a threonine dehydrogenase (TDH)-supported oxidation/decarboxylation of threonine [71]; *however, the open reading frame for TDH in humans has been identified as an inactive pseudo-gene* [72], in contrast to the retention of an active TDH in other mammals [73], [74], as well as bacteria [75]. In addition to aminoacetone and methylamine, other high *k*_cat_/K_m_ substrates warrant further investigation, namely dopamine and cysteamine. Neurons may be possible sources of dopamine since it is known that adipose tissue is innervated [76]. As mentioned previously, adipocytes are sensitive to dopamine through the β3-adrenoreceptor, which can play a role in insulin signaling. In addition, cysteamine is involved in the production of coenzyme A, an integral component of fatty acid breakdown and synthesis. From these kinetic studies, it is possible that AOC3 could have a novel regulatory role in both fatty acid metabolism and insulin signaling.

The murine 3T3-L1 cell line has enabled us to compare the properties of cell-associated AOC3 to that of purified enzyme. Given the ectopic property of the active site of cell-associated AOC3, the turnover of amines can be studied relatively easily via the detection of the extracellular peroxide produced in response to addition of primary amines. Mature adipocytes show uniform distribution across the plasma membrane of adipocytes (Fig. 3) and generate a linear production of hydrogen peroxide over a period of 30 min. The data in Table 4 show a reasonable agreement among K_m_ values determined using either whole cells or purified enzymes.

One of the surprising findings from this work is the demonstration that AOC3 catalyzes the oxidation of amines that are not produced at significant levels by human tissue: these include the branched chain aliphatic amines and aminoacetone. With regard to the former, high levels of isoamylamine accumulation (3.9 mM versus K_m_ of 4.5 mM for human AOC3) have been demonstrated in the media from overnight cultures of *Proteus morganii*, a human bacterial pathogen [77]. Other human pathogens that include *Bacteroides fragilis*, *Salmonella typhimurium*, *Yersinia entercolitica*, *Escherichia coli*, and *Clostridium perfringens* [78], [79] are also known to secrete branched chained amines, as well as small aliphatic amines (up to 2.3 mM n-propylamine and 250 uM n-butylamine). Thus, another possible role for the adipocyte-associated AOC3 is the detection of colonizing bacteria via their production of branched chain amines and aminoacetone. The production of H_2_O_2_ by bacterial-exposed adipocytes would be capable of impeding bacterial growth directly or possibly via the recruitment of immune cells. The location and stationary nature of adipocytes in subcutaneous tissue, which is often one of the first tissues infected after a wound [80] could rationalize the presence of an enzymatic reaction that works in concert with immune cells to minimize local proliferation of bacteria.

**Figure 4:**
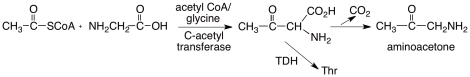
Condensation of acetyl-CoA and glycine to form the intermediate, 2-amino 3-ketobutyrate, a precursor of aminoacetone formation. Threonine dehydrogenase (TDH) normally catalyzes the reduction of 2-amino 3-ketobutyrate to form threonine and prevents its buildup; however, TDH is an inactive pseudo-gene in humans.

In closing, we also comment on a possible link of the adipocyte-associated AOC3 to the clinical symptoms of obesity [81], noting that there are several routes that could increase AOC3 activity, leading to an accompanying inflamed state. These include a possible change in gut-associated bacteria toward strains producing aliphatic amines [82], together with the lipid overload expected to accompany obesity and/or the increased availability of acetyl CoA seen in diabetes [83]. The latter states could enhance aminoacetone production via the condensation of acetyl CoA with glycine to produce 2-amino 3-ketobutyrate [84], Fig. 4. Although glycine C-acetyl transferase normally works in concert with TDH to reduce 2-amino 3-ketobutyrate to threonine in many microorganisms and animals [71], the absence of a functional TDH in humans [72] would exacerbate the accumulation of the immediate product, 2-amino 3-ketobutyrate [85], and its breakdown product (aminoacetone) in obese patients. The resulting turnover of aminacetone by AOC3, and the accompanying production of hydrogen peroxide, may then function as one of the triggers for the high macrophage recruitment and inflammation that occurs in obese adipose tissue.

## Supporting Information

Figure S1 **Alignment using MultAlin with identical amino acids in black and differences in red.** The murine enzyme is 83% identical and 91% similar to the human form. TPQ results from a post-translational modification at Y471.
(TIFF)

Figure S2 **Connolly surface comparison of amine oxidase active sites.** Comparison of size of active site “funnels” for substrate binding to the human AOC3 (A) and to a well-established methylamine oxidase from *H. polymorpha* (B) and Active site copper bound to its histidine ligands can be visualized at the bottom of each figure as a frame of reference. The cofactor to which substrate binds is immediately above the copper site.
(TIF)
