# Supplementary figures and images for: Implication for Functions of the Ectopic Adipocyte Copper Amine Oxidase (AOC3) from Purified Enzyme and Cell-Based Kinetic Studies
Source: PLoS One. 2012 Jan 4;7(1):e29270. doi: 10.1371/journal.pone.0029270 (PMC3251558; doi:10.1371/journal.pone.0029270)

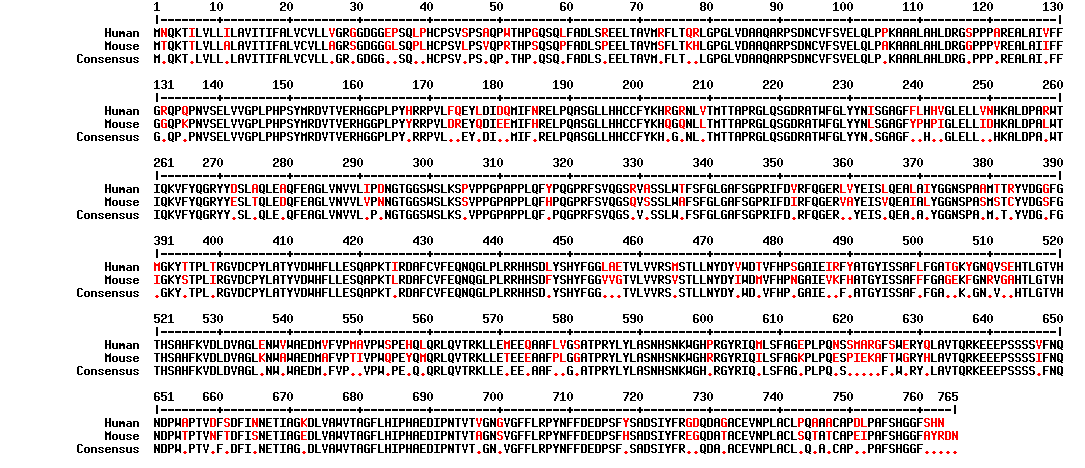

Supplement: Figure S1 — Alignment using MultAlin with identical amino acids in black and differences in red. The murine enzyme is 83% identical and 91% similar to the human form. TPQ results from a post-translational modification at Y471. (TIFF) [file pone.0029270.s001.tiff]

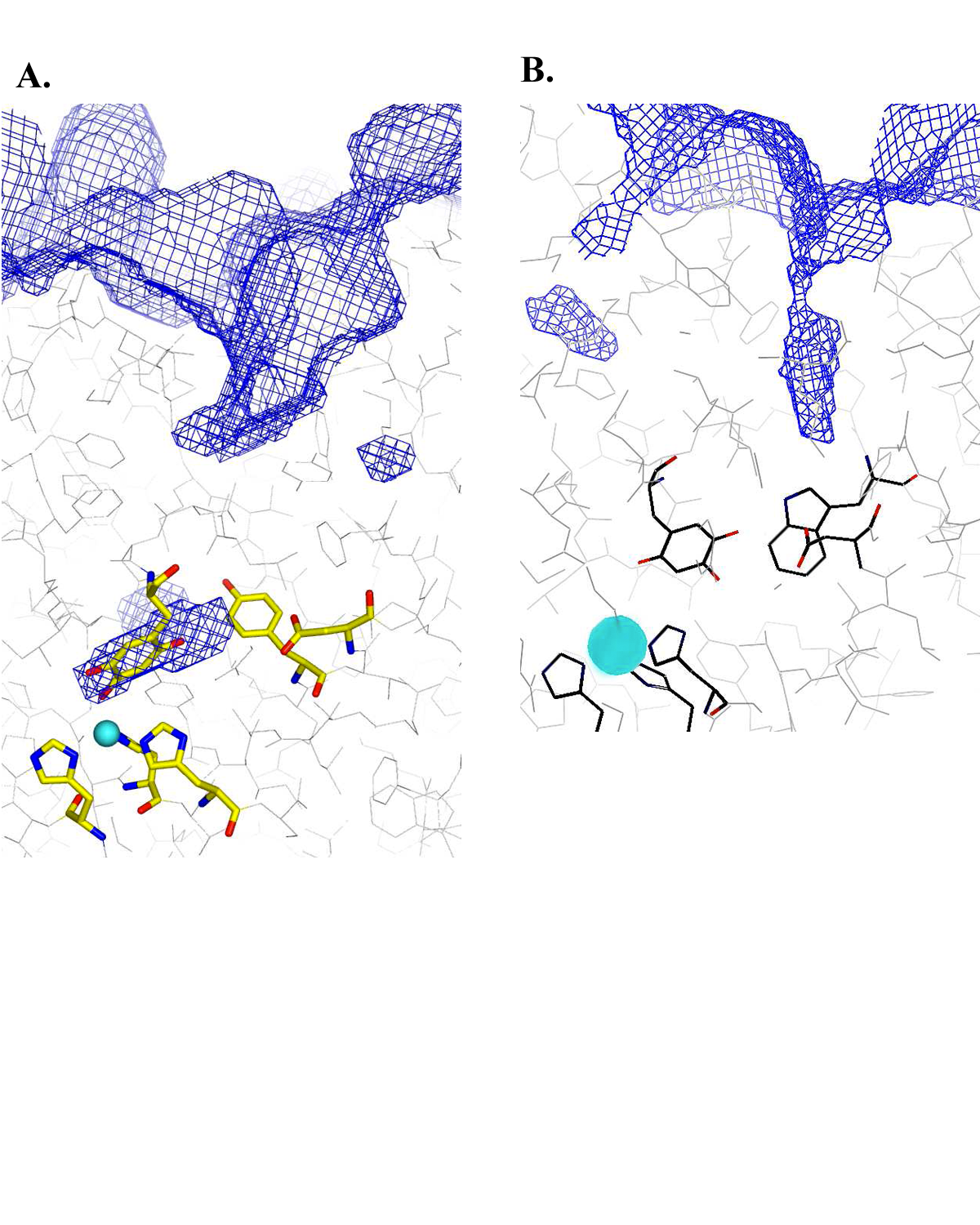

Supplement: Figure S2 — Connolly surface comparison of amine oxidase active sites. Comparison of size of active site “funnels” for substrate binding to the human AOC3 (A) and to a well-established methylamine oxidase from H. polymorpha (B) and Active site copper bound to its histidine ligands can be visualized at the bottom of each figure as a frame of reference. The cofactor to which substrate binds is immediately above the copper site. (TIF) [file pone.0029270.s002.tif]
